# NETO1 Guides Development of Glutamatergic Connectivity in the Hippocampus by Regulating Axonal Kainate Receptors

**DOI:** 10.1523/ENEURO.0048-17.2017

**Published:** 2017-07-03

**Authors:** Ester Orav, Tsvetomira Atanasova, Alexandra Shintyapina, Sebnem Kesaf, Michela Kokko, Juha Partanen, Tomi Taira, Sari E. Lauri

**Affiliations:** 1 Neuroscience Center, University of Helsinki, Helsinki FI-00014, Finland; 2 Department of Biosciences, University of Helsinki, Helsinki FI-00014, Finland; 3 Department of Veterinary Biosciences, University of Helsinki, Helsinki FI-00014, Finland

**Keywords:** auxiliary subunit, development, GluK1, hippocampus, kainate receptor, NETO

## Abstract

Kainate-type glutamate receptors (KARs) are highly expressed in the developing brain, where they are tonically activated to modulate synaptic transmission, network excitability and synaptogenesis. NETO proteins are auxiliary subunits that regulate biophysical properties of KARs; however, their functions in the immature brain are not known. Here, we show that NETO1 guides the development of the rodent hippocampal CA3-CA1 circuitry via regulating axonal KARs. NETO deficiency reduced axonal targeting of most KAR subunits in hippocampal neurons in a subtype independent manner. As an interesting exception, axonal delivery of GluK1c was strongly and selectively impaired in the *Neto1* ^−/−^, but not *Neto2* ^−/−^, neurons. Correspondingly, the presynaptic GluK1 KAR activity that tonically inhibits glutamate release at immature CA3-CA1 synapses was completely lost in the absence of NETO1 but not NETO2. The deficit in axonal KARs at *Neto1* ^−/−^ neurons resulted in impaired synaptogenesis and perturbed synchronization of CA3 and CA1 neuronal populations during development *in vitro*. Both these *Neto1* ^−/−^ phenotypes were fully rescued by overexpression of GluK1c, emphasizing the role of NETO1/KAR complex in development of efferent connectivity. Together, our data uncover a novel role for NETO1 in regulation of axonal KARs and identify its physiological significance in development of the CA3-CA1 circuit.

## Significance Statement

Kainate-type glutamate receptors (KARs) are highly expressed in the developing brain where they modulate synaptic transmission and synaptogenesis. NETO proteins act as auxiliary subunits for KARs; however, their functions at the immature circuit are not known. Using a combination of cell biology and electrophysiology in *Neto1*- and *Neto2*-deficient mouse models, we show that NETO proteins regulate axonal delivery of KARs. The deficit in axonal KAR at immature *Neto1* ^−/−^ neurons manifested as loss of presynaptic KAR activity and resulted in impaired synaptogenesis and perturbed development of the hippocampal CA3-CA1 circuit. These findings suggest novel roles for the NETO auxiliary subunits in orchestrating development and refinement of synaptic connectivity, a process increasingly implicated in developmentally originating neurologic disorders.

## Introduction

Ionotropic glutamate receptors are key signaling elements mediating and modulating fast excitatory neurotransmission in the brain. During development, glutamate receptors contribute to refinement of synaptic connectivity by triggering signals that regulate synaptic structure and function in response to patterned neuronal activity. In particular, the kainate-type glutamate receptors (KAR) are highly expressed in developing neuronal networks and have been implicated both in synaptogenesis (Tashiro et al., 2003; Marchal and Mulle, 2004; Vesikansa et al., 2007; Lanore et al., 2012; Sakha et al., 2016,) and activity-dependent plasticity at immature synapses (Lauri et al., 2006; Sallert et al., 2009; Clarke et al., 2014).

KARs are tetrameric receptors composed of five different subunits, GluK1-GluK5. In the hippocampus, GluK1 subunit containing receptors act presynaptically to modulate glutamate release (Lauri et al., 2001; Clarke et al., 2014; reviewed by Pinheiro and Mulle, 2008) and at interneurons, to regulate GABAergic transmission (reviewed by Lerma and Marques, 2013; Akgül and McBain, 2016). During the developmental fine-tuning of the circuitry, GluK1 subunit containing KARs are tonically activated by ambient extracellular glutamate (Lauri et al., 2005, 2006; Segerstråle et al., 2010). At immature CA3-CA1 synapses, tonically active presynaptic KARs inhibit glutamate release probability and, consequently, account for substantial frequency-dependent facilitation of synaptic transmission (Lauri et al., 2006). The frequency-dependent tuning of the transmission via presynaptic KARs is thought to be critical for appropriate development of the CA3-CA1 circuitry (Vesikansa et al., 2007; Hanse et al., 2009).

NETO proteins are auxiliary subunits that critically modulate the biophysical properties of KARs (reviewed by Copits and Swanson, 2012; Tomita and Castillo, 2012). Their roles in modulating the physiological functions of KARs have been studied at mossy fiber synapses in the area CA3, where NETO1 is responsible for the slow kinetics of postsynaptic KAR-mediated currents (Straub et al., 2011; Tang et al., 2011). In addition, both NETO1 and NETO2 may regulate synaptic recruitment of KARs (Copits et al., 2011; Tang et al., 2011, 2012; Wyeth et al., 2014; Sheng et al., 2015; Palacios-Filardo et al., 2016), although the exact molecular mechanisms of the NETO-dependent targeting of various KAR subunits remain unclear.

Here, we have studied the roles of NETO1/2 proteins in the developing glutamatergic circuitry. We show that both NETO1 and NETO2 are expressed in the developing hippocampus and that NETO1, but not NETO2, regulates axonal and presynaptic KARs at the CA3-CA1 circuit. NETO1 deficiency resulted in impaired synaptogenesis and disrupted synchronization of the CA3-CA1 neuronal populations. Both these *Neto1* ^−/−^ phenotypes were fully rescued by overexpression of GluK1c KAR subunit, consistent with a role of NETO1/KAR complex in development of efferent connectivity (Lauri et al., 2006; Vesikansa et al., 2012; Sakha et al., 2016).

## Materials and Methods

### Animals

Experiments were performed using wild-type (WT), *Neto1* ^−/−^, and *Neto2* ^−/−^ (C57Bl/6NCrl) mice (Ng et al., 2009; Tang et al., 2011) of either sex. All experiments with animals were done in accordance with the University of Helsinki Animal Welfare Guidelines.

### RT-PCR

Total RNA was purified from hippocampal tissue or dispersed WT hippocampal neurons using RNeasy Micro kit (QIAGEN) with an on-column DNase digestion step. cDNA was synthesized from 1 µg of total RNA using RevertAid First Strand cDNA synthesis kit (Thermo Scientific) with oligo(dT)_18_ primer. Real-time quantitative PCR (RT-qPCR) was performed using CFX96 Maxima SYBR Green qPCR Master Mix (Thermo Scientific) according to manufacturer’s recommendation and Real-Time PCR Detection System (Bio-Rad). All samples were analyzed in triplicate using *Gadph* as a reference gene and primers listed in Table 1. Initial copy number of a sample was obtained by relating the Ct of the sample to a standard curve plot. Relative quantification of *Neto* expression at different developmental stages was analyzed using standard 2^−ddCt^ method. The values are expressed as percentage (%) of postnatal day (P)4 (100% = gene expression level at P4).

**Table 1.** RT-qPCR primers

Target	Forward	Reverse
*Neto1*	TCATAGAAGCTGCCCCAAGG	AAGCCAAAGGGTCCATCTCG
*Neto2*	TTTGGAAGCTGCTCCTCGTC	TCCAAGTGATCAAACCGGCA
*Gapdh*	CAGTGCCAGCCTCGTCTCATA	TGGTAACCAGGCGTCCGATA

Reverse transcription (RT-PCR) was performed using Phusion High-Fidelity DNA polymerase (F-530L, Thermo Scientific) according to manufacturer’s recommendation to test *Neto1* and *Neto2* expression in the dispersed hippocampal neuron culture. PCR products were visually confirmed by gel electrophoresis.

### *In situ* hybridization (ISH)

ISH was conducted on 5-µm-thick paraffin sections as described previously (Wilkinson and Green, 1990) using digoxigenin-labeled antisense and sense RNA probes against *Neto1* and *Neto2* (Table 2). TSA-Plus Cyanine3/Fluorescein System (PerkinElmer) was used to visualize ISH signal, followed with a standard DAPI staining. The specificity of reaction conditions was tested using the sense RNA probes. At least three sections from two independent experiments were analyzed for each group. Samples were imaged using a Zeiss Axioimager M2 microscope with 20× objective and Axiocam HRc camera. For analysis, images with equal exposure time were imported to ImageJ software and the mean optical density of the *in situ* staining was normalized to the number of DAPI-positive cells in the analyzed area.

**Table 2.** Primers used for RT-PCR and for synthesis of ISH probes against *Neto1* and *Neto2*

Target	Forward	Reverse
*Neto1*	TGAGTTTGAGATGGGCGGCC	ACTGGTGTTGGTCAGCTGAT
*Neto2*	CTGATGGAATAGTGCGGTCT	GATCGTCCCATGAGTCTTCG
*Gapdh*	CAACGACCCCTTCATTGACC	AGTGATGGCATGGACTGTGG

### Plasmid generation and lentiviral vectors

Epitope tagged plasmid constructs for NETO1 and NETO2 were generated as described previously (Vesikansa et al., 2012). Briefly, *Neto1* and *Neto2* were amplified by PCR from rat brain cDNA and inserted into pTZ57R/T cloning vector (Thermo Scientific), and an in-frame HA tag was added to C terminus of NETO1 and 2 from Addgene plasmid 10792 (a gift of William Sellers). The epitope tagged constructs were subcloned into lentiviral transfer vector under a CMV promoter. Plasmids encoding various KAR subunits were described previously (Vesikansa et al., 2012; Sakha et al., 2016). The lentiviral particles were prepared as described previously (Vesikansa et al., 2012).

### Cell culture

Dispersed hippocampal neuron culture was prepared from P0 to P2 old mice pups. Hippocampi were isolated, digested with papain (500 μg/ml, Sigma) and triturated. Cells were plated with a density of 10,000 cells/µm on poly-L-lysin (Sigma) coated 24-well plates containing glass coverslips and grown in Neurobasal A (Gibco) medium containing 2% B27 supplement, 0.5 mM L-glutamine, and 1% penicillin/streptomycin (all from Life Technologies). At 3 d *in vitro* (DIV3), the cell cultures were infected with lentiviral vectors (Table 3) and fixed at DIV14 using 4% PFA in PBS.

**Table 3.** Lentiviral constructs used in this study

Protein	Promoter	Tag	Tag location
GFP	CMV	—	—
GluK1b(Q)	CMV	Flag	N terminus, after signal sequence
GluK1c(Q)	CMV	Flag	N terminus, after signal sequence
GluK2(Q)	CMV	myc	N terminus, after signal sequence
GluK4	CMV	myc	N terminus, after signal sequence
GluK5	CMV	myc	N terminus, after signal sequence
NETO1	CMV	HA	C terminus
NETO2	CMV	HA	C terminus

### Immunofluorescence

For immunostainings, fixed neurons were permeabilized with 0.2% Triton X-100 and incubated with PBS based blocking solution containing 5% goat serum, 1% bovine serum albumin (BSA), 0.1% gelatin, 0.1% Triton X-100, and 0.05% Tween 20. Primary antibodies [guinea pig anti-synaptophysin (1:2000; 101004, Synaptic systems), mouse anti-flag (1:1000; F1804, Sigma), rabbit anti-myc (1:1000; 06-549, Millipore), chicken anti-MAP2 (1:8000; AB5543, Millipore), and mouse anti-HA (1:1000; MMS-101R, Covance)] were diluted in a solution containing 1% BSA and 0.1% gelatin. The following secondary antibodies were used: goat anti-mouse Alexa Fluor 647 (1:2000; A21236, Life Technologies), goat anti-rabbit Alexa Fluor 647 (1:2000; A21245, Life Technologies), goat anti-guinea pig Alexa Fluor 568 (1:2000; A11075, Life Technologies), and goat anti-chicken Alexa Fluor 405 (1:500; ab175674, Abcam). The stained samples were mounted on microscope slides using Prolong Gold antifade reagent (P36934, Life technologies). Confocal images were acquired using a LSM Zeiss 710 confocal microscope (alpha Plan-Apochromat 63×/1.46 OilKorr M27 objective).

### Image analysis

NETO-HA (blue; Alexa Fluor 647) and synaptophysin (red, Alexa Fluor 568) colocalization was analyzed by calculating Pearson’s correlation coefficient (PCC) using Coloc2 plugin in Fiji (ImageJ) software. PCC was calculated for each z-stack separately. Axonal processes were identified using MAP2 staining. Cells exhibiting one MAP2 negative axon and several MAP2-positive dendrites were included in the analysis. Axonal intensity of the staining was measured using SynD/MATLAB (Schmitz et al., 2011) and normalized to intensity at the cell soma. For Syn puncta counting, only isolated axons without MAP2-positive dendritic contact were included in the analysis. Synaptophysin puncta (minimum size, 0.07 µm^2^) were counted using SynD and presented per 100 µm of axon length. All images were coded and analyzed in a blind fashion. Brightness/contrast and levels were adjusted in Photoshop for final figures.

### Organotypic slice culture

For preparation of organotypic cultures, hippocampi were dissected from P6-P7 day old WT or *Neto1* ^−/−^ mice in ice cold Gey’s balanced salt solution (GBSS; Sigma) supplemented with glucose (6.5 mg/ml). The hippocampi were mounted on 2% liquid agarose and 400-µm-thick slices were cut using a McIlwain tissue chopper. After 1 h recovery in carbogenated (95% O_2_ and 5% CO_2_) GBSS, the slices were transferred to MED64-probe plates (MED-P515A, Alpha Med Scientific) coated with polyethylenimine solution (0.1% PEI in 25 mM borate buffer) or onto Millicell-CM 0.4-μm membrane inserts (Millipore, Bedford) in six-well plates. The slices were cultivated in 250 µl of culture medium [Neurobasal A (Gibco), 2% B27-supplement (Gibco), 2 mM L-glutamine, and chloramphenicol] on MED64 probes or 1.2 ml of culture medium on the membrane inserts. Cultures were maintained in humidified CO_2_ incubator (+35°C), where MED64 probes were kept on a rocking stage. The culture media was changed every second day. In some of the WT and *Neto1* ^−/−^ slices, lentiviral particles encoding for GFP and GluK1c under separate syn1 promoters (Vesikansa et al., 2012) or GFP only were injected into the CA3 pyramidal region at DIV1. Slices grown on MED64 probes were subjected to microelectrode array recordings, while slices grown on membrane inserts were used for miniature EPSC (mEPSC) recordings at DIV5-DIV7. After recordings, the slices were fixed with 4% PFA and imaged to visualize slice morphology and infection rate in CA3 pyramidal layer.

### Electrophysiology

Acute parasagittal hippocampal sections (400 μm) were prepared from brains of neonatal (P4-P6) or juvenile (P14-P16) mice using standard methods (Lauri et al., 2006). Whole-cell patch clamp recordings were done from CA1 pyramidal neurons in acute or cultured slices using Cs-based intracellular solution containing 130 mM CsMeSO_4_, 10 mM HEPES, 0.5 mM EGTA, 4 mM Mg-ATP, 0.3 mM Na-GTP, 5 mM QX-314, and 8 mM NaCl; 285 mOsm, pH 7.2. Uncompensated series resistance (Rs < 30 MOhm) was monitored, and cells were discarded if Rs varied more that 20%. EPSCs were evoked by afferent stimulation with a basal frequency of 0.05 Hz in the presence of picrotoxin (100 μM) and D-AP5 (50 µM) to antagonize GABA_A_- and NMDA receptors, respectively. For mEPSC recordings, 1 µM TTX was also included. Data were collected using Axoscope 9.2 (Molecular Devices) or WinLTP software (Anderson and Collingridge, 2007). mEPSCs were analyzed with MiniAnalysis 6.0.3 program (Synaptosoft). Events were verified visually, and events with amplitude less than three times the baseline rms noise level were rejected. For time-course plots, detected events were calculated in 120-s bins. Evoked EPSC amplitude was analyzed using WinLTP. Decay time of the AMPA currents was calculated from 90–37% of the peak amplitude using the Mini Analysis Program 6.0.7.

### Microelectrode array recordings

The spontaneous activity of the slices was recorded for a 15-min period at DIV2 and/or DIV6 using the MED 64-amplifier (MED-A64HE1, Alpha MED Scientific). The data were collected with the Mobius Software (Alpha MED Scientific) using a low cut frequency of 0.1 Hz, high cut frequency of 5000 Hz and a sampling rate of 20 kHz. For analysis, the data were exported to LabView environment and filtered using Savitzky-Golay filter (half-width = 10 side points). Spikes were detected using the LabView Peak Detector algorithm (quadratic fit on a window of width = 3 points). The spike detection threshold was calculated using the median absolute deviation–based method (Chan et al., 2008) with a multiplicative factor *p* = 3.5, and verified visually. Bursts were defined as a sequence of spikes with a minimum of three spikes with a maximum interspike interval of 10 ms. For calculation of spike time tiling coefficient (STTC), at least two CA3 and CA1 channel pairs separated by at least 300 µm were selected for each slice. Time stamps for detected spikes from 10s period with clearly visible activity and without long (>300 ms) bursts were exported to MatLab. STTC was calculated as described previously (Cutts and Eglen, 2014), and the values for all the channel pairs in each slice were averaged and considered as *n* = 1.

### Statistical analysis

All statistics were calculated on raw (not normalized) data using Sigma Plot software. Shapiro-Wilk test was used to test for normal distribution and Student’s paired *t* test, one-way ANOVA with Holm-Sidak *post hoc* comparison or Kruskal-Wallis test was then used accordingly. All data are presented as mean ± SEM; *p* < 0.05 was considered statistically significant. In figures, the significance levels are indicated by asterisks as follows: *p < 0.05, **p < 0.01, ***p < 0.001.

## Results

### *Neto1* and *Neto2* are expressed in the neonatal hippocampus and may localize to presynaptic release sites

**Figure 1.**
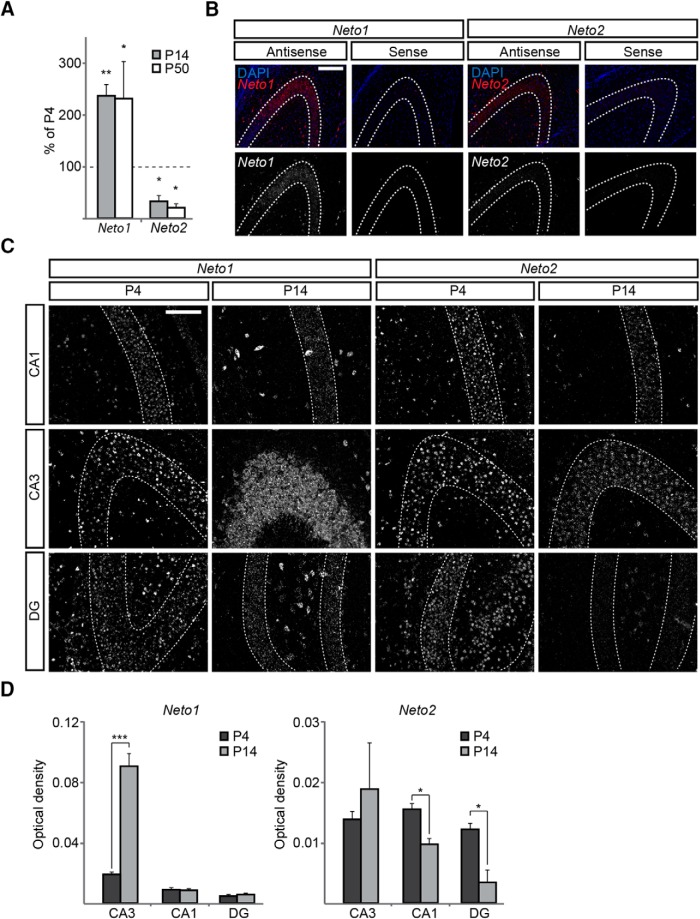
Developmental expression pattern of *Neto1* and *Neto2* in the hippocampus. ***A***, RT-qPCR analysis of *Neto1* and *Neto2* mRNA expression in the hippocampus. Data from three independent samples/group is represented as a percentage of the level at P4. ***B***, Example images showing staining with sense and antisense RNA probes against *Neto1* and *Neto2* in hippocampal sections. For analysis, the intensity of the ISH stain (red) is normalized to the number of cells within the analyzed region, identified with the DAPI stain (blue; upper row). The lower panels show the ISH stain as grayscale image. Dashed lines delineate the principal cell body layer. Scale bar, 100 µm. ***C***, Example images illustrating the ISH with antisense *Neto1* and *Neto2* probes at P4 and P14 hippocampal sections. Dashed lines show the principal cell layer. Scale bar, 100 µm. ***D***, Quantified data on the mean intensity of the ISH stain for *Neto1* and *Neto2* in areas CA1, CA3, and DG at P4 and P14 (*n* = 3–4 samples/group).

The expression of NETO proteins has been characterized in the adult brain, where NETO1 is strongly expressed in hippocampal CA3 pyramidal neurons (Michishita et al., 2003, 2004; Ng et al., 2009; Straub et al., 2011), whereas NETO2 is predominant in most brain regions except the hippocampus (Straub et al., 2011). As expression of both *Neto1* and *Neto2* in brain is developmentally regulated (Michishita et al., 2004), we first used RT-qPCR to quantify the expression of *Neto1* and *Neto2* mRNAs in hippocampus during early postnatal development. At P4, both *Neto1* and *Neto2* were expressed in hippocampus (estimated copy numbers 929 ± 87 and 1517 ± 106/0.1 µg of RNA for *Neto1* and *Neto2* transcripts, respectively, *n* = 3 for both groups, *p* = 0.005). During development, *Neto1* expression was strongly upregulated, in contrast to *Neto2*, which was downregulated, resulting in prevalent expression of *Neto1* in the juvenile (P14) and adult (P50) hippocampus (Fig. 1*A*,*B*). ISH detected both *Neto1* and *Neto2* in CA3 pyramidal neurons at P4 (Fig. 1*C*, see Fig. 1*B* for characterization of the ISH probes). *Neto2* was also expressed in the principal cells of other hippocampal regions, whereas the level of *Neto1* in CA1 pyramidal neurons and dentate granule cells was low, similar to that observed previously in the adult hippocampus (Michishita et al., 2003; Ng et al., 2009; Straub et al., 2011). Later in development (P14), *Neto2* expression remained in CA3 but was low in DG and CA1, while *Neto1* expression in CA3 region was strongly upregulated (Fig. 1*C*,*D*). In addition to pyramidal neurons, *Neto1* was strongly expressed in cell bodies in stratum radiatum, putatively representing GABAergic interneurons.

**Figure 2.**
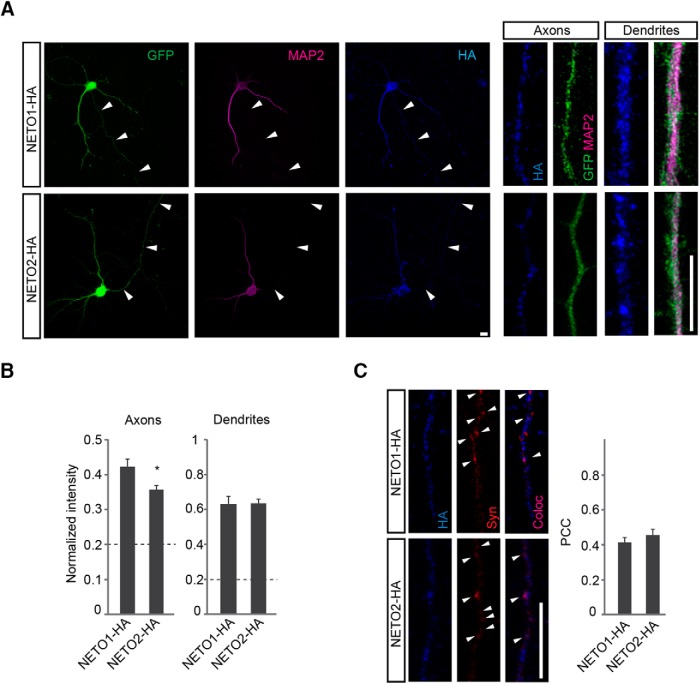
Subcellular localization of recombinant NETO proteins in hippocampal neurons. ***A***, Example images of hippocampal neurons expressing recombinant NETO1-HA and NETO2-HA (blue). MAP2 (purple) negative axons are marked with arrowheads. Cell morphology is visualized with GFP (green) expression. Scale bar, 10 µm. ***B***, Quantified data demonstrating delivery of lentivirally expressed NETO1-HA and NETO2-HA (blue) to MAP2-positive (purple) dendritic (NETO1-HA *n* = 31, NETO2-HA *n* = 39) and MAP2-negative axonal (NETO1-HA *n* = 27, NETO2-HA *n* = 26) processes in cultured hippocampal neurons. For quantification, the signal intensity in the neurites is normalized to the soma intensity. Scale bar, 10 µm. ***C***, Example images and quantified data demonstrating colocalization of lentivirally expressed NETO1-HA (*n* = 27) and NETO2-HA (*n* = 26; blue) with presynaptic marker synaptophysin (Syn) (red). Scale bar, 10 µm.

Both NETO1 and NETO2 have been shown to concentrate on postsynaptic fractions (Ng et al., 2009; Zhang et al., 2009; Straub et al., 2011; Tang et al., 2011); however, whether they also localize presynaptically in axons is not known. To investigate their subcellular distribution, HA-tagged NETO1 and NETO2 were overexpressed in WT mouse hippocampal neurons using lentiviral vectors. NETO1-HA was detected in MAP2 negative axonal processes (Fig. 2*A*,*B*) where it partially colocalized with synaptophysin (Fig. 2*C*). Also NETO2-HA was detected in MAP2 negative neurites and at axonal synaptophysin puncta; however, the relative axonal intensity of NETO2-HA was significantly lower as compared with NETO1-HA (Fig. 2*B*, *p* = 0.011). Both NETO1-HA and NETO2-HA were abundantly transported to MAP2-positive dendrites (Fig. 2*A*,*B*).

Together, these data indicate that both *Neto1* and *Neto2* are expressed in the immature hippocampus but show distinct region specific expression patterns, with *Neto1* being mainly restricted to CA3 and *Neto2* showing broader expression in principal neurons within all the hippocampal fields. Both proteins, and in particular NETO1, localize to axons and colocalize with synaptophysin-positive putative vesicle release sites when overexpressed in hippocampal neurons.

### NETO1 and NETO2 regulate axonal delivery of KAR subunits

**Figure 3.**
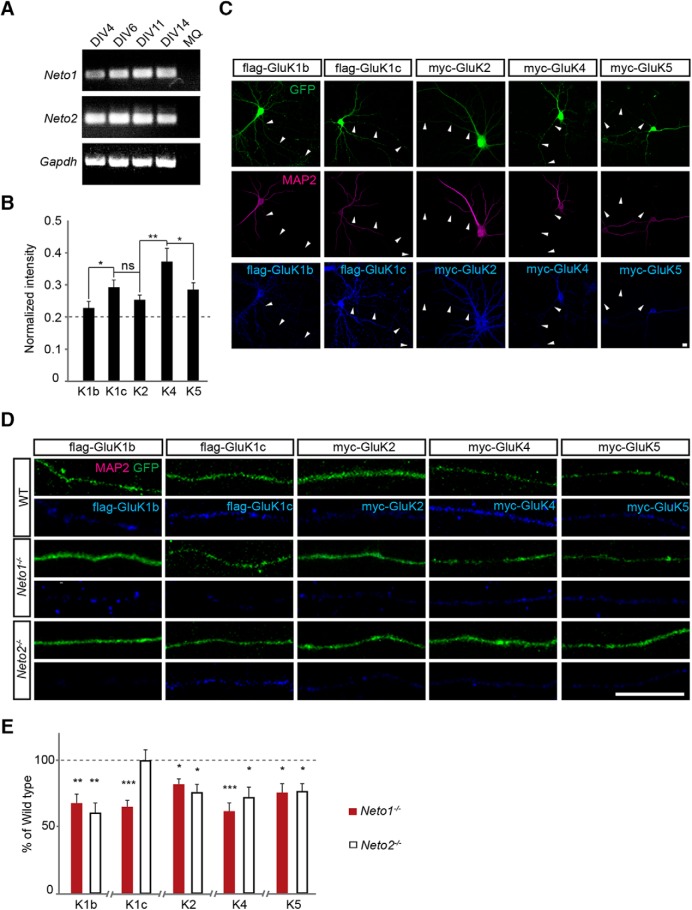
Loss of NETO1 leads to impaired GluK1c delivery to distal axons. ***A***, RT-PCR data depicting *Neto1*, *Neto2*, and housekeeping gene *Gapdh* expression in WT dispersed hippocampal neuron culture throughout the culture period (DIV4, DIV6, DIV11, and DIV14). ***B***, Quantified data on the delivery of recombinant KAR subunits to distal axons in WT hippocampal neurons. K1b: flag-GluK1b (*n* = 45), K1c: flag-GluK1c (*n* = 24), K2: myc-GluK2 (*n* = 39), K4: myc-GluK4 (*n* = 30), and K5: myc-GluK5 (*n* = 41). Signal intensity in distal axons is normalized to intensity in cell soma. ***C***, Example images of WT neurons expressing recombinant flag-GluK1b, flag-GluK1c, myc-GluK2, myc-GluK4, or myc-GluK5 (blue). MAP2 (purple) negative axons are marked with arrowheads. Cell morphology is visualized with GFP (green) expression. Scale bar, 10 µm. ***D***, Example images illustrating delivery of various recombinant KAR subunits (blue) to distal (>150 µm from soma) MAP2 negative axons in WT, *Neto1* ^−/−^, and *Neto2* ^−/−^ neurons. Scale bar, 10 µm. ***E***, Averaged data comparing KAR subunit delivery to WT versus *Neto1* ^−/−^ or *Neto2* ^−/−^ axons. K1b *n* = 47 and *n* = 24, K1c *n* = 13 and *n* = 29, K2 *n* = 41 and *n* = 34, K4 *n* = 18 and *n* = 22, K5 *n* = 34 and *n* = 48 for *Neto1* ^−/−^ and *Neto2* ^−/−^, respectively. Data are presented as % of corresponding values in the WT axons.

NETO proteins have been shown to regulate targeting of KARs to postsynaptic sites (Copits et al., 2011; Tang et al., 2011; 2012; Wyeth et al., 2014; Sheng et al., 2015; but see Straub et al., 2011), while no corresponding data for axons exist. In the absence of selective antibodies, myc- or flag-tagged recombinant KAR subunits were overexpressed in cultured hippocampal neurons from WT, *Neto1* ^−/−^, and *Neto2* ^−/−^ mice, to study the effect of NETO1 and NETO2 on axonal targeting of KARs. Two C-terminal splice variants of the GluK1, GluK1b and GluK1c, were included in the analysis because previous data indicates that these are differentially transported to axons (Vesikansa et al., 2012). RT-PCR indicated that both *Neto1* and *Neto2* were expressed in WT neurons under our culture conditions (Fig. 3*A*).

In WT neurons, all recombinant KAR subunits studied were detected in distal MAP2 negative axons (>150 µm from the soma), with flag-GluK1c and myc-GluK4 showing the highest relative intensity in this region (Fig. 3*B*,*C*; see also Vesikansa et al., 2012). When expressed in the *Neto1* ^−/−^ and *Neto2* ^−/−^ cultures, the labeling of most recombinant KAR subunits in the distal axons was lower as compared with WT neurons, suggesting a generic role for NETOs in KAR targeting. As an exception, the relative axonal intensity of flag-GluK1c in *Neto2* ^−/−^ cultures was comparable to WT, in indicating that the axonal delivery of GluK1c was selectively impaired in the absence of NETO1, but not NETO2 (Fig. 3*D*,*E*).

Together, these data suggest that NETO proteins promote trafficking of KAR subunits to axons. Interestingly, axonal delivery of GluK1c, the subunit that is highly expressed in the CA3 pyramidal neurons in the neonatal hippocampus (Vesikansa et al., 2012) was selectively impaired in the *Neto1* ^−/−^, but not *Neto2* ^−/−^, mice.

### Tonic activation of presynaptic KARs at immature CA3-CA1 synapses is not observed in the absence of NETO1

**Figure 4.**
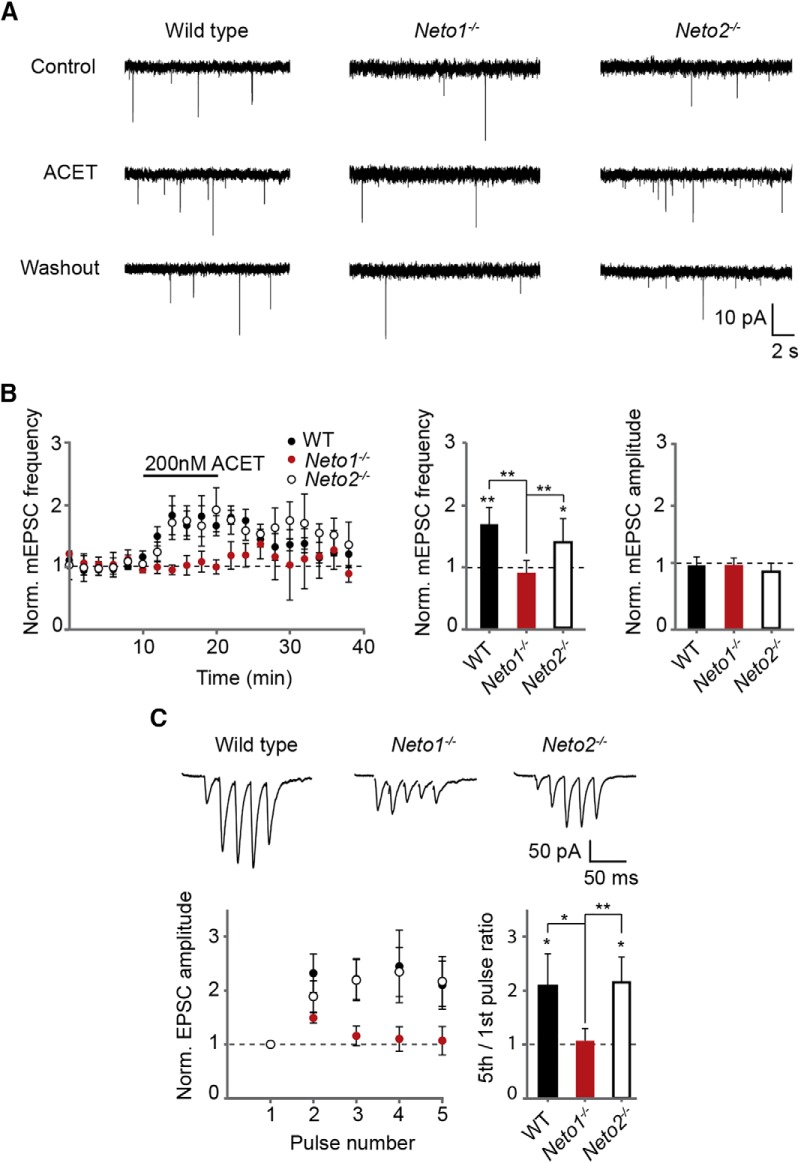
NETO1 is required for tonic activity of KARs at CA3-CA1 synapse in the neonatal hippocampus. ***A***, Example traces illustrating recording of mEPSCs from CA1 pyramidal neurons in WT, *Neto1* ^−/−^, and *Neto2* ^−/−^ slices (P5), under control conditions and in the presence of ACET (200 nM). ***B***, time-course plots and averaged data demonstrating the effect of ACET (200 nM) on mEPSC frequency in WT (*n* = 7) and *Neto2* ^−/−^ (*n* = 5), and *Neto1* ^−/−^ (*n* = 7) slices (P4-P6). ***C***, Example traces and pooled data demonstrating short-term facilitation of EPSCs in response to five-pulse 50-Hz stimulation in WT (*n* = 7) and *Neto2* ^−/−^ (*n* = 8), but not in *Neto1* ^−/−^ (*n* = 14) slices (P4-P6).

To study the role of NETO auxiliary subunits in regulation of presynaptic KAR function, patch clamp recordings of mEPSCs were made from CA1 pyramidal neurons in acute hippocampal slices from neonatal (P4-P6) WT, *Neto1* ^−/−^, and *Neto2* ^−/−^ mice. At immature CA3-CA1 synapses, presynaptic GluK1 subunit containing KARs are tonically activated by ambient glutamate to inhibit transmitter release (Lauri et al., 2006). Thus, application of a GluK1 selective antagonist enhances transmission, observed as an increase in the frequency of mEPSCs (Lauri et al., 2006; Sallert et al., 2009). As expected, application of ACET (200 nM) led to a significant increase in the mEPSC frequency (168 ± 27%, *n* = 7, *p* = 0.04) but had no effect on their amplitude (103 ± 13%) in the WT slices. A similar increase in mEPSC frequency was also observed in the *Neto2* ^−/−^ slices (140 ± 15%, *n* = 5, *p* = 0.01), in contrast to *Neto1* ^−/−^ slices where ACET had no significant effect on mEPSCs (83 ± 19%, *n* = 7; Fig. 4*A*,*B*). The basal mEPSC frequency was significantly lower in both *Neto1* ^−/−^ (*p* = 0.04), and *Neto2* ^−/−^ (*p* = 0.006) slices as compared with WT, while the mEPSC amplitude was lower in *Neto2* ^−/−^ (*p* = 0.02), but not in *Neto1* ^−/−^ slices (frequency 0.17 ± 0.02, 0.10 ± 0.01, and 0.10 ± 0.01 Hz; amplitude 16.2 ± 1.2, 16.7 ± 0.8, and 13.6 ± 1.1 pA for WT, *Neto1* ^−/−^, and *Neto2* ^−/−^, respectively).

The tonic inhibition of glutamate release by GluK1 subunit containing KARs also manifests as facilitation of transmission during high-frequency activity at immature CA3-CA1 synapses (Lauri et al., 2006). Consistent with a loss of tonic KAR activity, facilitation of EPSCs in response to five-pulse 50-Hz afferent stimulation was not observed in CA1 pyramidal neurons in *Neto1* ^−/−^ slices (5th/1st EPSC ratio 1.05 ± 0.22, *n* = 14), in contrast to WT and *Neto2* ^−/−^ slices (WT 2.10 ± 0.58, *n* = 7; *Neto2* ^−/−^ 2.16 ± 0.46, *n* = 8; Fig. 4*C*). No differences in the kinetics of the AMPA EPSCs, evoked by single pulse afferent stimulation, were detected between the genotypes (decay_90-37%_ WT 4.7 ± 0.74 ms, *n* = 6; *Neto1* ^−/−^ 4.05 ± 0.72 ms, *n* = 8, p _WT,_ *_Neto1_*_−/−_ = 0.44 and *Neto2* ^−/−^ 3.63 ± 0.23 ms, *n* = 6, p _WT,_ *_Neto2_*_−/−_ = 0.17; data not shown). These data show that *Neto1* deficiency impairs the tonic activation of presynaptic KARs at the immature synapses.

### Absence of tonic KAR activity in *Neto1^−/−^* slices is due to loss of functional presynaptic KARs

**Figure 5.**
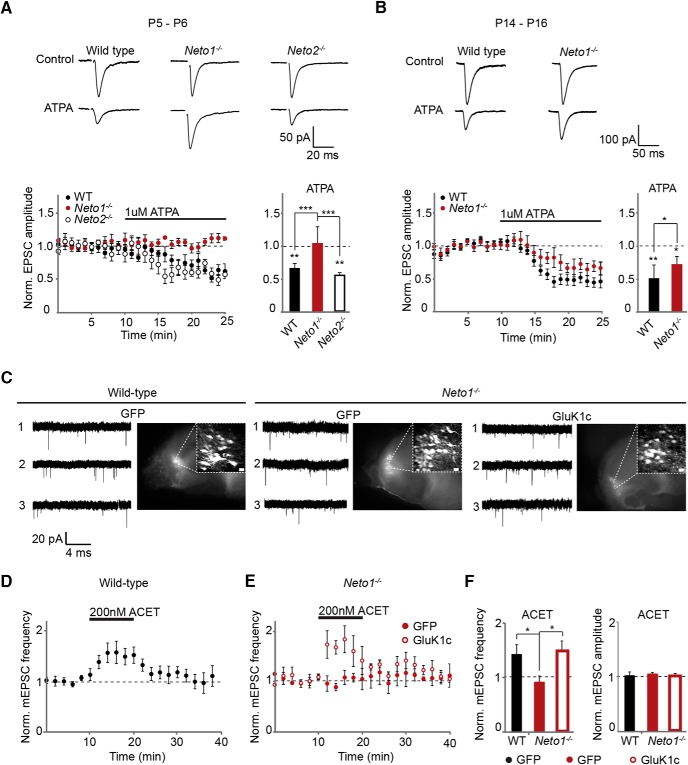
NETO1 deficiency leads to loss of functional presynaptic KARs at immature CA3-CA1 synapses that is rescued with GluK1c overexpression in the area CA3. ***A***, Example traces and pooled data showing that ATPA (1 µM) decreases synaptic transmission at CA3-CA1 synapse in WT (*n* = 6) and *Neto2* ^−/−^ (*n* = 7), but not in *Neto1* ^−/−^ (*n* = 9) acute slices (P4-P6). ***B***, Example traces and pooled data showing the effect of ATPA (1 µM) on EPSCs in two week old (P14-P16) WT (*n* = 7) and *Neto1* ^−/−^ (*n* = 8) acute slices. ***C***, Example traces of mEPSCs recorded from CA1 pyramidal neurons in WT and *Neto1* ^−/−^ organotypic cultures expressing GFP or GluK1c in CA3 pyramidal layer, under control conditions (trace 1), in the presence of ACET (200 nM; trace 2), and during washout (trace 3). Images show the GFP or GluK1c/GFP expression in the CA3 pyramidal layer in the corresponding cultures (scale bar, 20 µm). ***D***, Time-course plot demonstrating the effect of ACET (200 nM) on mEPSC frequency in WT organotypic cultures expressing GFP (WT *n* = 7) in area CA3. ***E***, Time-course plot demonstrating the effect of ACET (200 nM) on mEPSC frequency in *Neto1* ^−/−^ organotypic cultures expressing GFP (*n* = 5) or GluK1c (*n* = 4) in area CA3. ***F***, Pooled data for the experiments depicted in ***D***, ***E***.

NETO interaction increases the agonist sensitivity of GluK1 KARs (Fisher, 2015; Li et al., 2016). Thus, the simplest explanation for the loss of tonic KAR activity in the *Neto1* ^−/−^ slices is that in the absence of NETO1, the presynaptic receptors cannot be activated by the ambient glutamate due to their lower affinity. To address this possibility, we tested the effect of ATPA, a selective agonist for GluK1 KARs (Clarke et al., 1997, Clarke and Collingridge, 2002) on transmission at the CA3-CA1 synapses. ATPA binds to GluK1 with a high affinity and activates GluK1 subunit containing recombinant KARs at submicromolar concentrations in the absence of NETO proteins (Jane et al., 2009). Thus, at 1 µM concentration, ATPA is expected to activate GluK1 subunit containing KARs in NETO-deficient neurons. Application of ATPA (1 µM) led to a significant decrease in the amplitude of evoked EPSCs in both WT (66 ± 7% of control, *n* = 6, *p* = 0.007) and *Neto2* ^−/−^ mice (EPSC amplitude 55 ± 3% of control, *n* = 7, *p* = 0.002), but had no effect on transmission at *Neto1* ^−/−^ slices at P4-P6 (99 ± 23%, *n* = 9, *p* = 0.1; Fig. 5*A*). Therefore, loss of tonic KAR activity at immature CA3-CA1 synapses of *Neto1* ^−/−^ mice reflects absence of functional presynaptic KARs and cannot be explained merely by reduced glutamate affinity of GluK1 subunit containing receptors.

The subunit composition as well as the downstream signaling mechanism of the presynaptic KARs changes during development (Sallert et al., 2007; Vesikansa et al., 2012). To study whether NETO1 and NETO2 regulate presynaptic KAR function later on in development, we tested the effect of ATPA at two weeks (P14-P16) old mice, when KARs are no longer tonically active. At this developmental stage, ATPA application resulted in a decrease in the EPSC amplitude also in the *Neto1* ^−/−^ mice (77 ± 7%, *n* = 8, *p* = 0.03); however, this effect was significantly (*p* = 0.03) smaller to that observed at the WT (53 ± 7%, *n* = 7; Fig. 5*B*). Furthermore, no significant differences in the frequency or amplitude of mEPSCs were detected between the genotypes at P14-P16 [frequency 0.42 ± 0.05, 0.37 ± 0.04, and 0.41 ± 0.04 Hz; amplitude 16.6 ± 0.7, 16.4 ± 0.8, and 17.8 ± 0.4 pA for WT (*n* = 14), *Neto1* ^−/−^ (*n* = 10), and *Neto2*^−/−^ (*n* = 10), respectively; data not shown]. These data suggest that NETO1 deficiency affects presynaptic KAR function predominantly during early postnatal development, when GluK1 subunit is highly expressed at CA3 pyramidal neurons and presynaptic KARs are physiologically activated.

NETO deficiency reduced the axonal delivery of KAR subunits but did not completely block it. Therefore, we reasoned that also NETO independent mechanisms for KAR targeting exist and overexpression of KAR subunits could rescue KAR levels in NETO-deficient axons. To test this possibility, we made organotypic cultures from WT and NETO1-deficient mice and used lentiviral vectors to overexpress GluK1c or GFP in the CA3 pyramidal cells. Patch clamp recordings of mEPSCs were made from CA1 pyramidal neurons at DIV5-DIV7. Similar to the acute slices from neonatal hippocampus, ACET (200 nM) application in the GFP-expressing WT slice cultures was associated with an increase in the frequency of mEPSCs (143 ± 17%, *n* = 7, *p* = 0.03) but no change in their amplitude (102 ± 6%). ACET application had no effect on mEPSC frequency in GFP-expressing *Neto1* ^−/−^ cultures (92 ± 10%, *n* = 5), while a significant increase in mEPSC frequency was observed in *Neto1*^−/−^ cultures where GluK1c was overexpressed in the CA3 pyramidal cells (148 ± 0.18%, *n* = 4, *p* = 0.03; Fig. 5*C–F*). Thus, overexpression of GluK1c in the CA3 pyramidal neurons was sufficient to rescue the tonic presynaptic KAR activity in the *Neto1* ^−/−^ slices. Together, these data support the conclusion that the loss of tonic KAR activity in the *Neto1* ^−/−^ mice is due to loss of axonal GluK1 subunit containing KARs.

### Absence of NETO1, but not NETO2, affects presynaptic differentiation in hippocampal neurons

**Figure 6.**
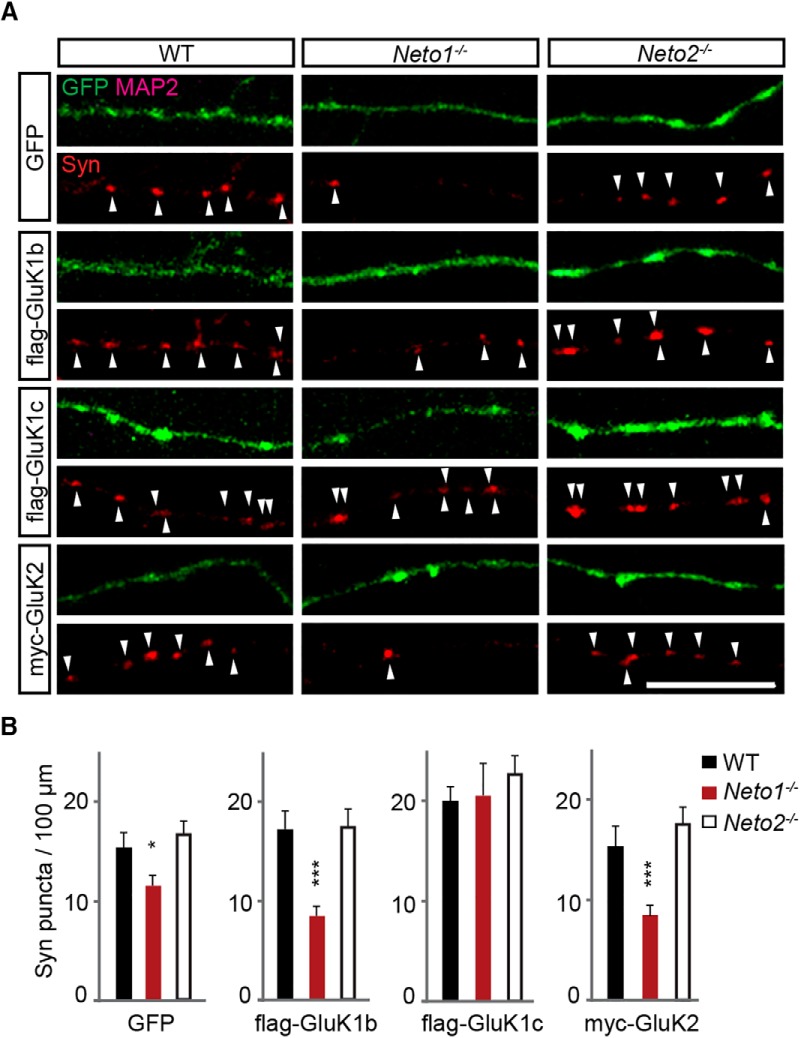
Loss of axonal GluK1c in *Neto1* ^−/−^ neurons results in reduced density of synaptophysin immunopositive (Syn) puncta. ***A***, Example images of Syn puncta (red) in MAP2 negative WT, *Neto1* ^−/−^, and *Neto2* ^−/−^ axons with lentiviral expression of GFP, GFP + flag-GluK1b, GFP + flag-GluK1c, or GFP + myc-GluK2. For clarity, the blue channel with flag/myc staining is not shown. Scale bar, 10 µm. ***B***, Quantification of the Syn puncta (red) density in the three genotypes with lentiviral expression of GFP (WT *n* = 31; *Neto1* ^−/−^ *n* = 36; *Neto2* ^−/−^ *n* = 28), GFP + flag-GluK1b (WT *n* = 28; *Neto1* ^−/−^ *n* = 29; *Neto2* ^−/−^ *n* = 24), GFP + flag-GluK1c (WT *n* = 28; *Neto1* ^−/−^ *n* = 19; *Neto2* ^−/−^ *n* = 31), or GFP + myc-GluK2 (WT *n* = 40; *Neto1* ^−/−^ *n* = 30; *Neto2* ^−/−^ *n* = 27).

The obtained results show that *Neto1* ^−/−^ mice lack presynaptic KAR activity at the CA3-CA1 circuit and suggest this is due to impaired axonal targeting of GluK1 subunit containing KARs. Presynaptic KARs have been implicated in development of the synaptic connectivity between CA3 and CA1 pyramidal neurons (Lauri et al., 2006; Sallert et al., 2009; Vesikansa et al., 2007, 2012; Sakha et al., 2016). Therefore, we went on to examine the density of synaptophysin puncta in WT, *Neto1* ^−/−^, and *Neto2* ^−/−^ axons, focusing only on MAP2 negative processes with no visible postsynaptic (MAP2 positive) contacts. The mean density of synaptophysin puncta in GFP-expressing *Neto1* ^−/−^ (77 ± 7% of WT, *n* = 36, *p* = 0.04), but not *Neto2* ^−/−^ (108 ± 9% of WT, *n* = 28), axons was significantly lower as compared with WT (Fig. 6).

In WT and *Neto2* ^−/−^ neurons, overexpression of flag-GluK1c increased the density of synaptophysin puncta in isolated axons (WT 136 ± 12%, *n* = 19, *p* = 0.007; *Neto2* ^−/−^ 135 ± 11%, *n* = 31, *p* = 0.01) as previously shown (Sakha et al., 2016). In *Neto1* ^−/−^ neurons, however, this increase was significantly larger (179 ± 26%, *n* = 19, *p* < 0.001), resulting in similar density of the synaptophysin puncta as in GluK1c-expressing WT axons. In contrast, overexpression of flag-GluK1b or myc-GluK2 had no significant effect on the density of axonal synaptophysin puncta in any of the genotypes (Fig. 6*A*,*B*). These data suggest that the lack of axonal KARs, likely containing the GluK1c subunit, impairs presynaptic differentiation in the *Neto1* ^−/−^ neurons.

### NETO1 regulates development of functional connectivity in the CA3-CA1 circuit

**Figure 7.**
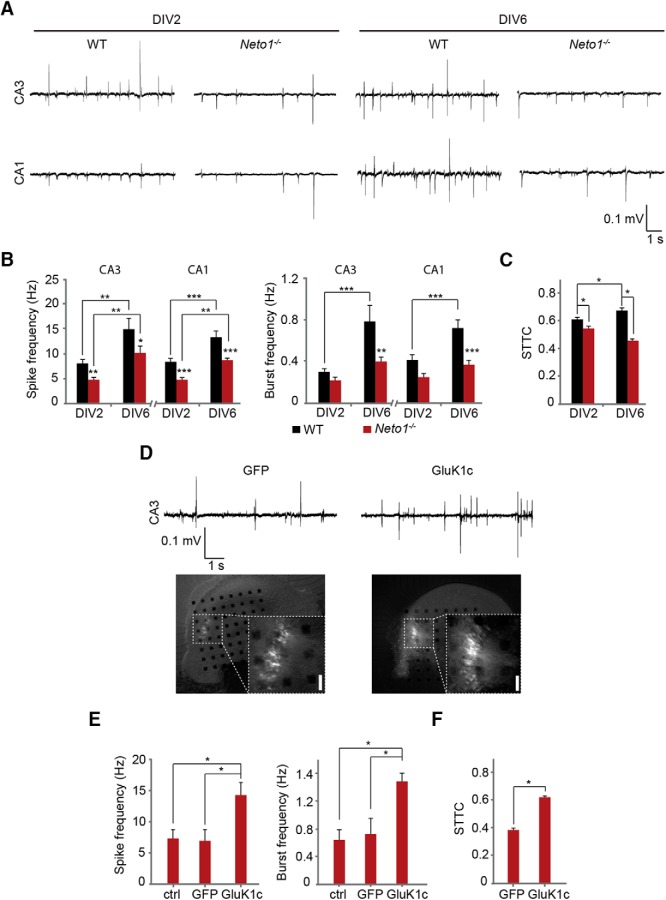
Neto1-dependent axonal targeting of GluK1c is required for developmental synchronization of the CA3-CA1 circuit. ***A***, Example traces illustrating spontaneous activity in CA3 and CA1 regions of WT and *Neto1* ^−/−^ hippocampal slices cultured on MEA probes at DIV2 and DIV6. ***B***, Quantification of spike frequency and burst frequency in WT (DIV2 *n* = 12, DIV6 *n* = 11) and *Neto1* ^−/−^ (DIV2 *n* = 12, DIV6 *n* = 13) slices. Data are averaged from at least two channels located in CA3 and in CA1 pyramidal regions/slice. ***C***, Averaged data for STTC_CA3,CA1_ measuring temporal correlation of spiking activity between CA3 and CA1 subregions in *Neto1* ^−/−^ (*n* = 12) and WT (*n* = 11) slices. ***D***, Images showing GFP and GluK1c/GFP expression in CA3 principal cells in *Neto1* ^−/−^ slices. Example traces demonstrate the activity in the virally transduced region of the corresponding slice cultures at DIV6. Scale bar, 150 µm. ***E***, Quantification of spike frequency and burst frequency for nonmanipulated (ctrl, *n* = 7), GFP (*n* = 7)-, or GluK1c/GFP (*n* = 8)-expressing CA3 cell populations in *Neto1* ^−/−^ slices at DIV6. ***F***, Analysis of the STTC_CA3,CA1_ in *Neto1*^−/−^ (DIV6) slices were the CA3 cell populations have been virally transduced to express GFP (*n* = 7) or GluK1c/GFP (*n* = 8).

To address the physiologic significance of NETO1 in the development of CA3-CA1 circuitry, hippocampal slices from WT and *Neto1* ^−/−^ mice were cultured on microelectrode array (MEA) probes and the spontaneous network activity was recorded at DIV2 and DIV6 using channels located at the CA3 and CA1 pyramidal cell regions. During this period in culture, the network activity patterns in WT slices gradually transformed from asynchronous firing (DIV2) to highly correlated bursting activity that ultimately takes the form of ictal-like epileptiform bursts (DIV6 onwards; data not shown). Thus, in the WT slices, the mean frequency of population spikes and bursts (defined as at least three events with interspike-interval of <10 ms) increased during development *in vitro* both in areas CA3 and CA1 (Fig. 7*A*,*B*). In the *Neto1* ^−/−^ slices, the mean spike frequency was lower as compared with WTs at both developmental stages in CA1 and in CA3 (Fig. 7*A*,*B*). At DIV2, the mean frequency of bursts in *Neto1* ^−/−^ cultures was not different from the WTs. However, as there was no significant increase in bursts during development, the burst frequency in the NETO1-deficient slices was significantly lower as compared with WTs at DIV6 (Fig. 7*A*,*B*).

The observed differences in the overall activity patterns might be mediated by a variety of neural mechanisms operating at different cell types within the hippocampal network. To focus on the CA3-CA1 connectivity, we went on to analyze temporal correlation in spiking activities of the CA3 and CA1 neuronal populations. Correlation in the CA3-CA1 population spiking activity was assessed with spike time tiling coefficient (STTC_CA3,CA1_; Cutts and Eglen, 2014), which is independent on the differences in basal firing frequency. In WT cultures, STTC_CA3,CA1_ increased significantly during development from DIV2 to DIV6 (*n* = 11, *p* = 0.031; Fig. 7*A*,*C*). In *Neto1* ^−/−^ cultures, STTC_CA3,CA1_ was significantly lower than in WT cultures already at DIV2, and more evidently at DIV6 (Fig. 7*C*). Importantly, in *Neto1* ^−/−^ cultures STTC_CA3,CA1_ did not increase during development, suggesting that NETO1 affects development of synchrony between CA3-CA1 neuronal populations.

Given that synaptogenesis in *Neto1* ^−/−^ axons was dependent on GluK1c levels, we went on to test whether overexpression of GluK1c in the presynaptic CA3 neurons would rescue the observed defect in synchronization of CA3-CA1 circuit in the *Neto1* ^−/−^ cultures. Lentiviral overexpression of GluK1c in the CA3 pyramidal cells in *Neto1* ^−/−^ slice cultures was associated with a higher frequency of spikes and bursts as compared with controls at DIV6 (GFP-expressing or nonmanipulated CA3 neurons in the same slices; Fig. 7*D*,*E*). Interestingly, STTC_CA3,CA1_ for GluK1c-expressing *Neto1* ^−/−^ CA3 neuron populations was significantly higher as compared with GFP-expressing controls, and similar to WT slices (Fig. 7*F*). These data suggest that elevated expression of KARs in presynaptic neurons can fully rescue the defect in functional development of the CA3-CA1 connectivity in the *Neto1* ^−/−^ slice cultures.

## Discussion

NETO proteins were identified as auxiliary subunits for KARs originally in 2009 (Zhang et al., 2009; Tang et al., 2011); however, our knowledge on their physiologic significance in the brain is still limited. In particular, very little is known on the functions of NETOs in the developing brain where KARs are widely expressed. Here, we show that NETO1 affects development of the hippocampal CA3-CA1 circuit via regulating axonal KARs. In the absence of NETO1, functional presynaptic KARs at the nascent connections are lost, leading to impaired synaptogenesis. Thus, in addition to the previously characterized roles of NETO proteins in modulating postsynaptic KARs, we here demonstrate that NETO1 regulates presynaptic KARs in developing hippocampal networks and thereby contributes to wiring of the CA3-CA1 circuitry.

### NETO proteins promote axonal recruitment of KARs

Although presynaptic functions for KARs have been described in various areas of the brain, the mechanisms governing axonal targeting of KARs are not well understood. Our data using epitope tagged KAR subunits in cell cultures shows that NETO1 and NETO2 deficiency impairs axonal targeting of most KAR subunits to a similar extent, except for GluK1c whose axonal targeting was selectively reduced in *Neto1* ^−/−^, but not *Neto2* ^−/−^, neurons.

The observed generic and subunit-dependent effects of NETOs on KAR targeting resembles what has been previously shown for TARP and cornichon families of AMPA auxiliary subunits. These auxiliary proteins act as chaperones regulating assembly and ER export of AMPA receptors (Jackson and Nicoll, 2011; Straub and Tomita, 2011), a mechanism that could also explain the subunit independent effects of NETOs on KARs. Regarding axonal delivery of GluK1c splice variant, the specific regulation by NETO1, but not NETO2, may be achieved in different ways. For instance, GluK1c contains a C-terminal 29 amino acid insert that is not present in any of the other KAR subunits, which could mediate splice-variant specific protein interaction(s). Such proteins could affect NETO-GluK1c interaction directly or indirectly, for example by recruitment of second messengers enabling covalent modification. Indeed, it was recently shown that mutation of putative C-terminal phosphorylation site in NETO proteins disrupt NETO-dependent postsynaptic trafficking of GluK1 (Sheng et al., 2015). When overexpressed in cell cultures, both NETO1 and NETO2 were detected in axons, supporting the hypothesis that NETO1 associates with the KARs complex to directly regulate its axonal and presynaptic targeting. Unfortunately, in our hands, the available antibodies were not selective enough to study the subcellular localization of native NETO proteins in intact tissue to resolve whether they are localized at presynaptic terminals in the neonatal or adult brain.

### NETO1 is required for tonic presynaptic KAR activity at immature synapses

Synaptic transmission in neonatal NETO1-deficient slices was completely insensitive to GluK1 selective drugs, which are specific for presynaptic KARs at WT CA3-CA1 synapses (Lauri et al., 2006). Furthermore, frequency-dependent facilitation of transmission, attributed to tonic inhibition of release probability by presynaptic KARs at immature synapses (Lauri et al., 2006) was not observed in *Neto1* ^−/−^ slices, together indicating that NETO1 is critical for immature-type presynaptic KAR function.

GluK1c and GluK4 are the subunits previously implicated in tonic presynaptic KAR activity at the immature synapses (Vesikansa et al., 2012). Thus, GluK1c splice variant is coexpressed in the CA3 pyramidal cells with the subunit GluK4 during the first postnatal week temporally coinciding with the tonic KAR activity, after which its expression is rapidly downregulated (Vesikansa et al., 2012). Our ISH and RT-qPCR analysis indicates that both *Neto1* and *Neto2* mRNA are expressed in CA3 pyramidal neurons in the neonatal hippocampus. Both NETO1 and NETO2 affected axonal targeting of KARs and we cannot rule out the possibility that some of the NETO2 functions in the knockout mice are compensated for. Nevertheless, the loss of presynaptic KAR function at *Neto1* ^−/−^ CA3-CA1 synapses during the time GluK1c is expressed at CA3 pyramidal neurons corresponds well to the finding that NETO1 specifically regulates axonal recruitment of GluK1c in cultured neurons. Later on in development (P14) when GluK1c expression is already downregulated, presynaptic KARs could be pharmacologically activated to depress synaptic transmission also at the NETO1-deficient slices, although the effect was smaller as compared with the WTs. Finally, overexpression of GluK1c in the CA3 pyramidal neurons was able to rescue the presynaptic KAR function at CA3-CA1 synapses in the NETO1-deficient slices. Together, these findings show that NETO1 deficiency leads to loss of presynaptic KAR function particularly at immature synapses, where GluK1c is endogenously expressed. NETO1 promotes but is not absolutely required for presynaptic KARs function, as its dependence on NETO1 is reduced on KAR overexpression and during development.

NETO1 deficiency reduced the axonal levels of recombinant GluK1c to ∼60% of the control level, raising a question whether such a modest reduction in axonal targeting can explain the complete loss of functional presynaptic KAR at immature synapses. It is possible that the observed effects of NETOs on recombinant KARs underestimate the reliance of native KARs on auxiliary subunits, as the mechanisms responsible for subcellular segregation of KARs may be overloaded during elevated expression, leading to nonphysiological compartmentalization (Fièvre et al., 2016). On the other hand, compartmentalization of KARs in dissociated cultured neurons may be weak as compared with intact tissue, where diverse extracellular interactions take place and may regulate synaptic recruitment of KARs directly or indirectly (Fièvre et al., 2016; Matsuda et al., 2016). While our data show that NETO proteins promote axonal delivery of KAR subunits, it does not define the extent by which NETO1 affects targeting of endogenous KARs in native tissue environment. As such, we cannot exclude the possibility that NETO1 regulates presynaptic KAR function also by additional mechanisms, for example by contributing to the assembly of the signaling complex that mediates the effects of KARs on glutamate release or by altering the biophysical properties by which the receptors are activated by ambient glutamate.

### NETO proteins and synaptogenesis

Axonal KARs have been implicated in development and maturation of synaptic connectivity both in cultured neurons (Tashiro et al., 2003; Sakha et al., 2016) as well as in intact circuitry (Lauri et al., 2006; Vesikansa et al., 2012). Consistent with the loss of presynaptic KAR function, we observed that the density of axonal Syn puncta was reduced in *Neto1* ^−/−^, but not *Neto2* ^−/−^, neurons and fully rescued by GluK1c overexpression. These data support the hypothesis that NETO1 promotes presynaptic development during the time of intense synaptogenesis and circuit refinement via regulation of axonal GluK1 subunit containing KARs. Since our analysis of presynaptic puncta was done on isolated axons, these findings further confirm that axonal GluK1c containing KAR affects presynaptic differentiation directly, in the absence of postsynaptic contact. Intriguingly, in contrast to the present results in isolated axons, GluK2 overexpression led to a strong increase in Syn puncta in axons that grow in bundles in microfluidic grooves (Sakha et al., 2016). While the exact mechanism explaining this difference remains unknown, these results call for better understanding of the mechanisms and consequences of the GluK1 versus GluK2 extracellular interactions (Matsuda et al., 2016, Sheng et al., 2017).

Interestingly, although the density of presynaptic release sites was compromised selectively in the *Neto1* ^−/−^ axons, the basal mEPSC frequency at immature CA1 pyramidal cells was significantly lower in both *Neto1* ^−/−^ and *Neto2* ^−/−^ slices as compared with WT. The mEPSC frequency reflects the number and release probability (Pr) of functional synaptic inputs. Based on the lower facilitation of EPSCs during 50-Hz stimulation, mean Pr in the *Neto1* ^−/−^ mice is higher as compared with WTs, consistent with the loss of tonic KAR mediated inhibition of release (Lauri et al., 2006). Yet, the higher Pr in the *Neto1* ^−/−^ mice would compensate for any effects of a lower synapse density on mEPSC frequency. In *Neto2* ^−/−^, but not *Neto1* ^−/−^, slices, the mEPSC amplitude was lower as compared with the WT, pointing to a postsynaptic phenotype. NETO2 can target KARs to postsynaptic sites at CA1 neurons (Copits et al., 2011; Sheng et al., 2015), which might influence synaptogenesis (Marchal and Mulle, 2004; Lanore et al., 2012; Petrovic et al., 2017) via a different but complementary mechanism. In addition, NETO2 might influence synaptic maturation via KAR independent mechanisms, for example via the recently described interaction with the KCC2 (Ivakine et al., 2013) implicated in regulation of spine density (Li et al., 2007). The effects of NETO deficiency on mEPSCs were only observed during early postnatal development, indicating that any dependency of synaptogenesis on NETO proteins is not absolute and can be compensated by other mechanisms during development.

### NETO1 influences developmental synchronization of neuronal populations

The significance of the NETO1-dependent presynaptic KAR activity on the development of CA3-CA1 circuitry was further studied at the network level. To that end, NETO1-deficient slices were cultured on MEA probes allowing functional properties of the neuronal network to be monitored through development *in vitro*. During the first days *in vitro*, activity of CA3 and CA1 neuronal populations became increasingly synchronized, in parallel with an increase in synaptic connectivity in these cultures (De Simoni et al., 2003; Chiappalone et al., 2007). In the absence of NETO1, synchronization between CA3 and CA1 firing activities was significantly impaired. This phenotype was fully rescued by GluK1c expression at CA3 neurons, suggesting an important role of NETO1-dependent axonal KARs in functional development of the network.

Apart from the reduced synchronization, we observed reduced excitability (i.e. population spike frequency) in the NETO1-deficient neurons while GluK1c overexpression in the CA3 pyramidal neurons robustly increased CA3 population firing rate. Higher charge transfer through postsynaptic KARs is expected to amplify spike transmission due to their integrative properties on transmission (Frerking and Ohliger-Frerking 2002; Sachidhanandam et al., 2009; Pinheiro et al., 2013). Thus, the reduced firing rate in the absence of NETO1 likely reflects loss of postsynaptic KARs in the pyramidal neurons (Straub et al., 2011), leading to reduced postsynaptic integration and excitability. In cultured hippocampal neurons, a critical level of excitability is required for synchronization to take place (Penn et al., 2016). In *Neto1* ^−/−^ slices; however, some network bursts were generated already at DIV2, suggesting that the level of excitability was not limiting the entrainment of neurons to synchronous populations. In addition to pyramidal neurons, NETO1 expression was also detected at GABAergic interneurons, which have a critical role in network oscillations and synchrony. However, the reduced synchrony between CA3-CA1 neuronal populations in the *Neto1* ^−/−^ slices was fully rescued by overexpression of GluK1c at the CA3 pyramidal neurons, suggesting that weaker synaptic connectivity between CA3-CA1 neuronal populations due to loss of presynaptic KARs significantly contributed to disrupted synchrony in the NETO1-deficient slices.

There is strong evidence suggesting that correlation between neuronal spike times is vital for organization of the neural circuits during development (Xu et al., 2011). Despite the defect in developmental synchronization of the network, NETO1-deficient mice have no gross abnormalities in the hippocampal morphology, but show a defect in hippocampus-dependent learning paradigms (Ng et al., 2009). The lack of striking morphologic phenotype is not surprising given the high capacity of developing neuronal networks for compensatory plasticity (Huupponen et al., 2007; Yin and Yuan, 2015). However, it is possible that such compensatory mechanisms mask vulnerability that can be later on manifested, for example as an increased susceptibility for neurologic disorders (Charlesworth et al., 2016).

## Synthesis

Reviewing Editor: Christian Hansel, University of Chicago Decisions are customarily a result of the Reviewing Editor and the peer reviewers coming together and discussing their recommendations until a consensus is reached. When revisions are invited, a fact-based synthesis statement explaining their decision and outlining what is needed to prepare a revision will be listed below. The following reviewer(s) agreed to reveal their identity: Zafar Bashir, Manzoor Bhat

This manuscript by Lauri and colleagues reports on the role of Neto1 and Neto2 auxiliary proteins on kainate-type glutamate receptor (KAR) functional modulation and its contribution to establishing synaptic circuitry during early postnatal development. The Neto proteins associate with KARs to alter their biophysical properties and thus may contribute to early synaptic functions and allow neural circuits to form and mature. Questions have remained as to whether Neto proteins have functional redundancy and whether they play a selective role in KAR function and which KAR might be selectively involved or affected. This manuscript builds on extensive work of the Lauri lab in studying presynaptic KARs. In this report, Orav and colleagues examine the role auxiliary NETO proteins in the developmental regulation of axonal kainate receptors (KAR) at CA3-CA1 synapses in the hippocampus. The authors show that Neto1 plays a more prominent role in the development of the hippocampal CA3-CA1 circuitry by regulating axonal KARs as Neto1 deficiency reduced axonal targeting of most KAR subunits in hippocampal neurons in a subtype independent manner, and that KAR GluK1c was selectively affected in Neto1 deficient and not Neto2 deficient neurons. In addition, axonal delivery of GluK1c was strongly and selectively impaired in the Neto1 mutant but not Neto2 mutant neurons. Interestingly, Neto1 mutant phenotypes were rescued by overexpression of GluK1c allowing the authors to conclude that Neto1 experts it influence via GluK1c during early development. Overall the studies are well done but the manuscript has many issues that make it difficult to follow the main conclusions of the data presented.

The role of axonal kainate receptors in synapse function and organization have received abundant attention. However, the targeting and regulation of these presynaptic receptors is poorly understood. This manuscript helps fill this void in understanding by examining axonal KARs at CA3-CA1 synapses.

The manuscript follows a logical flow with well-reasoned experiments. The data is of good quality. The results should appeal to a focused audience on pre-synaptic receptor function.

The paper must be carefully revised. The “Discussion” section is highly redundant. It would be better to have subtitles to make meaningful points in the Discussion for readers to learn what the data sets convey and how Neto proteins modulate receptor functions during development.

Major comments:

1) Fig 3: Why is basal frequency of release lower in both NETO1 and NETO2 KOs? It is not clear why these experiments were only done in P4 and not in older animals.

2) In the results (P11) it is suggested that the phenotype of NETO1 KO cannot be due to reduced affinity. However, this suggestion needs some more explanation. Based on the data presented there is no reason why this cannot be down to reduced affinity.

3) Is there any difference in evoked EPSPs in NETO KOs compared to WT?

4) Fig 3c - the NETO2 KO trace does not look very representative of the pooled data.

5) 3e - why is ATPA tested in p14-16? This was not made clear. Why are there no data for p15 for NETO2 KOs?

6) Fig 6b - data averaged from at least 2 CA1 and 2 CA3 channels - explain the reasoning behind the averaging across CA3 and CA1

7) If NETO is required for CA3 terminal expression of KARs that influences CA3-CA1 activity what drives the increased firing frequency in CA3 itself?

8) What is the function of NETO1 in GABAergic interneurons (p9)? Does this play any role in the synchrony across CA3 regions?

9) Does loss of Neto1 affect the in vivo protein levels or distribution of Gluk1c? Is Neto1 required for the stability of GluK1c?

10) Does Neto1 substitute the function of Neto2 in Neto2 mutants? It is clear that only Neto1 mutants show a phenotype?

11) The in vitro cell culture data is not rigorous as most proteins overexpressed in cultures neurons often show co-localization?

12) Do Neto proteins affect post-synaptic localization of other GluR receptors? How specific is the presynaptic localization of Neto proteins?

13) As stated, “The defect in axonal delivery of GluK1c in the Neto1-/- but not Neto2-/- mice CORRELATES with selective loss of presynaptic KAR function in these mice.” This claim could be bolstered by more direct functional evidence. Because the authors use overexpression of GluK1c to overcome the Neto1-induced deficit in synaptophysin puncta, it should be possible to overcome the loss of KAR-dependent inhibition of mEPSC frequency (Figure 3A-B) using GluK1c overexpression. The authors should perform this experiment.

14) Both KARs/NETOs are expressed throughout hippocampal neurons including in their soma/dendrites (e.g., Figures 2A and 4C). NETO regulates the biophysical properties and targeting of KARs at these locations. Because the authors DO NOT specifically perturb these proteins in the axon, it is plausible that the developmental alterations in synaptic activity and circuit function in their Neto1 loss-of-function mutant may result from KAR/NETOs deficits in these other compartments (rather/or in combination with axonal deficits). This point should be discussed.

Minor points:

“Acutely prepared hippocampal slices” should be specified on line 229 (the authors also use dispersed neurons and slice cultures so it is an important distinction to make).

Overexpression of KARs should be emphasized throughout the manuscript in reference to viral transduction of KARs (i.e., lines 209, 296, and 325).
